# Improved guidance is needed to optimise diagnostics and treatment of patients with thyroid cancer in Europe

**DOI:** 10.1007/s12020-023-03610-5

**Published:** 2023-11-25

**Authors:** Christelle de la Fouchardière, Laura Fugazzola, Laura D. Locati, Clara V. Alvarez, Robin P. Peeters, Pilar Camacho, Iris M. Simon, Barbara Jarząb, Romana Netea-Maier

**Affiliations:** 1 Institut Paoli-Calmettes, Medical Oncology Department, Marseille, France; 2 Division of Endocrine and Metabolic Diseases, IRCCS Istituto Auxologico Italiano, 20145 Milan, Italy; 3 Department of Pathophysiology and Transplantation, University of Milan, 20122 Milan, Italy; 4 Department of Internal Medicine and Therapeutics, University of Pavia, Pavia, Italy; 5 Medical Oncology Unit, Istituti Clinici Scientifici Maugeri IRCCS, Pavia, Italy; 6 Neoplasia & Endocrine Differentiation, Centro de Investigación en Medicina Molecular y Enfermedades Crónicas (CIMUS), University of Santiago de Compostela (USC), 15782 Santiago de Compostela, Spain; 7 Instituto de Investigación Sanitaria (IDIS), 15706 Santiago de Compostela, Spain; 8 Department of Internal Medicine, Erasmus Medical Center, Rotterdam, The Netherlands; 9 Eli Lilly and Company, Indianapolis, IN USA; 10 Department of Nuclear Medicine and Endocrine Oncology, Maria Sklodowska-Curie National Research Institute of Oncology Gliwice Branch, 44-102 Gliwice, Poland; 11 Division of Endocrinology, Department of Internal Medicine, Radboud University Medical Center, 6525 GA Nijmegen, The Netherlands

**Keywords:** Thyroid cancer, Diagnosis, Guidelines, Molecular testing, Real-world evidence

## Abstract

Although thyroid cancer (TC) is generally associated with a favourable prognosis, there are certain high-risk groups with a clear unmet therapeutic need. Unravelling the genomic landscape of TC has recently led to the development of novel effective targeted treatments. To date, these treatments have mostly been evaluated in non-randomised single-arm phase II clinical trials and are consequently non-reimbursed in several countries. Furthermore, most of these agents must be tailored to individual patient molecular characteristics, a context known as personalised cancer medicine, necessitating a requirement for predictive molecular biomarker testing. Existing guidelines, both in Europe and internationally, entail mostly therapeutic rather than molecular testing recommendations. This may reflect ambiguity among experts due to lack of evidence and also practical barriers in availability of the preferred molecular somatic screening and/or targeted treatments. This article reviews existing European recommendations regarding advanced/metastatic TC management with a special focus on molecular testing, and compares findings with real-world practice based on a recent survey involving TC experts from 18 European countries. Significant disparities are highlighted between theory and practice related to variable access to infrastructure, therapies and expertise, together with the insufficient availability of multidisciplinary tumour boards. In particular, practitioners’ choice of what, how and when to test is shown to be influenced by the expertise of the available laboratory, the financing source and the existence of potential facilitators, such as clinical trial access. Overall, the need of a collaborative initiative among European stakeholders to develop standardised, accessible molecular genotyping approaches in TC is underscored.

## Introduction

Thyroid cancer (TC) is the most frequent malignancy of the endocrine system [1]. Although the estimated mortality risk is low overall (0.04–0.05%), there are certain high-risk subgroups with poor prognosis and a clear unmet therapeutic need [2, 3].

The molecular genotyping of TC has become particularly relevant since the recognition of targetable alterations led to the development of new therapeutic options. Molecular genotyping now offers the opportunity of personalised cancer medicine (PCM) for the subgroup of patients with aggressive oncogene-addicted TC (advanced/metastatic, radioiodine [RAI]-refractory TC) [2]. However, except for *Rearranged during Transfection (RET) gene mutation* assessment [4], there are no clear guidelines for molecular testing in TC to dictate which predictive markers to look for, at which timepoint and with which methodology [5]. It is also unclear whether biomarkers should be tested to determine likely prognosis for either adults or children with TC, which would then affect treatment strategy (e.g., extent of surgery and type of systemic treatment). Finally, there are cases where a targetable alteration may be identified, but the relevant drug is lacking formal approval for TC indications, as in the case of the proto-oncogene B-Raf (BRAF) protein inhibitor dabrafenib for BRAF-mutated locally advanced/metastatic anaplastic TC (ATC) in Europe.

This viewpoint summarises existing recommendations regarding advanced/metastatic TC management with a special focus on molecular testing and compares these with current real-world practices in Europe based on a recent survey of 86 practitioners in 18 European countries [6].

## Current European clinical practice guidelines in advanced/metastatic TC

Multikinase inhibitors (MKIs) targeting the vascular endothelial growth factor (VEGFR) were first approved by the European Medicines Agency (EMA) and the US Food and Drug Administration (FDA) for the treatment of advanced/metastatic TC [medullary TC (MTC) and non-MTC] in 2013. Although MKIs are still considered first-line systemic treatment for progressive, advanced/metastatic, RAI-refractory differentiated TC (DTC) and MTC, they are associated with significant toxicities [7–9].

Since 2013, several targeted agents have shown clinical value and emerged as new therapeutic options in this setting [10, 11]. Table 1 summarises current EMA-approved drugs for progressive, advanced/metastatic TC which is not amenable to locoregional therapy. However, availability issues (e.g., for vandetanib and cabozantinib) have been noted in some European countries.

**Table 1:** EMA-approved drugs for progressive, advanced/metastatic TC which is not amenable to locoregional therapy

Agent(s)	EMA-approved indication for adults	ESMO recommendation strength (Grade, Quality of evidence)	ESMO- MCBS v1.1 [39]	ESCAT score [40]
Sorafenib [41]	Progressive, advanced/metastatic, RAI-refractory DTC	I, A	2	N/A
Lenvatinib [42]	Progressive, advanced/metastatic, RAI-refractory DTC	I, A	2	N/A
Cabozantinib [43, 44]	Progressive, advanced/metastatic MTC	I, A	3	N/A
	RAI-refractory DTC progressing post treatment with sorafenib and/or lenvatinib	I, A	2	N/A
Vandetanib [45]	Progressive, advanced/metastatic MTC	I, A	2	N/A
Selpercatinib [46, 47]	Advanced *RET* fusion-positive TC following prior treatment with sorafenib and/or lenvatinib	V, B	3	I-B
	Advanced *RET*-mutant MTC who require systemic therapy^*^	V, B	3	I-B
Larotrectinib [48]	*NTRK* fusion-positive advanced/metastatic solid tumours (including TC) when no other satisfactory treatment options are available	V, B	3	I-C
Entrectinib [49]	*NTRK* fusion-positive advanced/metastatic solid tumours (including TC) when no prior treatment with NTRK inhibitor and no other satisfactory treatment options available	V, B	3	I-C

*DTC* differentiated thyroid cancer, *EMA* European Medicines Agency, *ESCAT* ESMO Scale for Clinical Actionability of molecular Targets, *ESMO* European Society for Medical Oncology, *MCBS* ESMO-Magnitude of Clinical Benefit Scale, *MTC* medullary thyroid cancer, *N/A* not applicable, *RAI* radioiodine, *TC* thyroid cancer

^*^As of 2022, no prior treatment with cabozantinib and/or vandetanib is required according to the Committee for Medicinal Products for Human Use (CHMP) opinion

In the case of locally advanced/metastatic ATC, which is associated with particularly poor prognosis, the European Society for Medical Oncology (ESMO) Clinical Practice Guideline (CPG) recommends upfront *BRAF V600E* mutation testing and, if positive, administration of the BRAF inhibitor dabrafenib plus the mitogen-activated protein kinase (MEK) inhibitor trametinib (IV, B; ESMO Scale for Clinical Actionability of molecular Targets [ESCAT] score: I-B). In the presence of druggable mutations other than *BRAF V600E* (e.g. *RET* fusions, neurotrophic tyrosine receptor kinase [*NTRK*] fusions), ESMO CPG proposes targeted therapies as a therapeutic option [10]. However, this would require upfront comprehensive testing beyond *BRAF V600E*. In addition, the EMA has not approved either the dabrafenib/trametinib combination or RET inhibitors for this indication to date, thus making the application of guidelines particularly problematic in many European countries [12].

In addition, disparities among national guidelines and ESMO recommendations are frequently observed, a fact that can be related not only to lack of regular updates but also to varying novel drug availability and access to testing in each European country [13]. For example, Spanish recommendations published in 2020 do not include RET inhibitors in either RAI-R DTC or MTC management, possibly because approval was lacking at the time of publication; BRAF inhibitors for ATC are mentioned in the context of clinical trials, with a comment on the lack of formal approval; and larotrectinib is placed in first-line treatment of *NTRK* fusion-positive advanced, progressive RAI-refractory DTC, which could be considered as a quite liberal translation of the current EMA approval (indicating NTRK inhibitors only when no other satisfactory treatment options are available) [14]. In the current updated Italian guidelines on TC, drug recommendations per TC type are lacking altogether [15]. In France, inclusion in clinical trials is recommended for patients with TC harbouring specific molecular alterations [5].

## What do European clinical practice guidelines say about molecular tests?

To incorporate novel systemic therapy developments for treating patients with advanced/metastatic TC, ESMO has published a recent CPG update [10]. However, this update focuses more on therapeutic rather than molecular testing guidelines.

Table 2 summarises ESMO recommendations on molecular testing. Generally, testing for targetable genetic alterations is suggested when a systemic therapy is considered, so as to allow for PCM [10, 11]. Testing for *BRAF V600E* mutation, *RET* and *NTRK* rearrangements is recommended for DTC and ATC; and *RET* mutations for MTC, with next-generation sequencing (NGS) as the preferred approach, if available. In MTC, allele-specific real-time polymerase chain reaction (PCR) is also recommended to detect *RET* mutations. However, the guidelines do not comment on the turnaround time of testing, which may be of critical importance for patients with highly aggressive tumours such as ATC. Also, they do not discuss whether molecular testing performed at diagnosis can be informative for therapeutic decisions at the time of disease progression/recurrence, or whether a new sample should be obtained and evaluated before targeted agent administration, probably due to lack of relevant evidence. Moreover, they do not specify the starting material (DNA vs RNA). A recent publication by the ESMO Precision Medicine Working Group recommends the use of circulating tumour DNA (ctDNA) for *BRAF V600E*, *RET* mutations and *NTRK 1/2/3* fusions if tissue is not available or when faster results are clinically required [16].

**Table 2:** Summary of ESMO Clinical Practice Guideline update recommendations on molecular testing in patients with advanced/metastatic TC [10]

Advanced/metastatic TC type	When to test	What to test	How to test	Recommendation strength (grade, quality of evidence)
DTC	When a systemic therapy is planned	*RET* and *NTRK* rearrangements	NGS, if available	III, C
ATC	In unresectable/metastatic disease	*BRAF V600E mutation, RET* and *NTRK* rearrangements	NGS, if available	-
MTC	When a systemic therapy is planned	*RET* mutations	Allelic-specific real-time PCR or NGS	III, A

*ATC* anaplastic thyroid cancer, *DTC* differentiated thyroid cancer, *MTC* medullary thyroid cancer, *NGS* next-generation sequencing, *PCR* polymerase chain reaction, *TC* thyroid cancer

National guidelines can be even less instructive about molecular testing in TC. For example, the current Spanish recommendations suggest somatic *RET* mutation testing in advanced/metastatic sporadic MTC, but make no mention of other targetable genetic lesions in DTC and ATC [14]. The updated 2022 Polish guidelines recommend testing for somatic alterations to identify molecular therapeutic targets in MTC, RAI-refractory DTC and ATC, but do not specify the optimal testing method [17], whereas the current Italian guidelines for TC make no recommendations on molecular testing [15].

To add further perplexity, the 2022 WHO Classification of Endocrine and Neuroendocrine Tumours, 5th edition, recommends immunohistochemistry (IHC) for the detection of *BRAF V600E* mutations, *RAS Q61R* mutations and *ALK* fusions and makes no comment regarding *RET* and *TERT* testing, despite recognising them as frequently altered genes in TC [18].

## Methodological considerations for molecular testing in TC

Defining an optimal molecular testing strategy in TC may be challenging; factors that should be considered involve not only technical aspects but also availability of methods related to cost [13]. As mentioned above, the ESMO recommendations favour NGS, possibly because it represents a specific, sensitive and (nearly) all-inclusive technique, which allows simultaneous detection of multiple genomic alterations and can obtain the most information from the least amount of sample [10]. However, NGS is not widely available or reimbursed. If panels are used to simultaneously test for the most frequent alterations, then infrequent alterations may be missed. Also, there are certain caveats related to the starting material (DNA or RNA). DNA NGS may detect fusions of unknown functional significance or fail to detect rearrangements involving large intronic regions (making it problematic for testing *NTRK2* and *NTRK3* rearrangements) [19]. An RNA-based assay, on the other hand, requires high-quality material (both the tested sample and the positive controls) and skilful preparation.

IHC and fluorescence in situ hybridisation (FISH) may be used for fusion detection and are widely available. Triaging samples by IHC to select for molecular *NTRK* testing has been suggested as a strategy to optimise workflow and reduce costs [20]. In fact, ESMO recommendations for *NTRK* fusion detection suggest either RNA-NGS or pre-screening by pan-TRK IHC followed by confirmatory RNA-NGS for unselected populations where *NTRK1/2/3* fusions are uncommon [19], such as those with TC. However, IHC lacks sensitivity and specificity for *RET* fusion detection and may have reduced sensitivity for *NTRK3* fusion detection [21]. For *RET* fusions, ESMO recommends NGS; if not available, FISH or reverse-transcription PCR (RT-PCR) is recommended [4]. Advantages and disadvantages of available techniques are well described in existing literature [21–25].

IHC and FISH are less expensive than NGS when testing a single alteration. However, NGS-based parallel testing for all actionable genetic aberrations may be more cost-efficient than single-gene–based sequential testing [26]. As a cost-saving strategy, Macerola et al., Elisei et al. and Haddad et al. proposed testing for *BRAF* and *RAS* genes, followed by further analyses in case of negative results [25, 27, 28]. Capdevila et al. recommended routine testing for genetic alterations as part of the clinical work-up for patients with RAI-refractory advanced/metastatic FTC, PTC and PDTC and for all patients with ATC using a versatile testing algorithm that accommodated most scenarios related to technical and availability issues. As part of this algorithm, rapid, low-cost IHC could be used as initial screening; however, even positive results need to be confirmed by RNA-NGS, FISH or RT-PCR [29]. Pre-screening with RT-PCR might represent an alternative approach, given its high specificity and sensitivity; however, it requires validated in-house protocols. Thus, laboratories with the appropriate expertise are essential. Of note, when pre-screening testing strategies are used, sample availability needs to be carefully considered. This also applies when investigating single alterations rather than multi-target molecular characterisation. Liquid biopsy (ctDNA) may be utilised as an alternative sample source when formalin-fixed, paraffin-embedded (FFPE) biopsy material is insufficient, but this methodology is not widely available [4, 30]. In addition, the feasibility and performance of circulating free RNA (cfRNA) on gene fusions is still unclear.

## Current real-world molecular testing practices in Europe: results from a collaborative survey

At present, there is no clear algorithm for molecular testing in TC that can be widely adopted. To investigate real-world practices in Europe regarding molecular genotyping in aggressive TC, a survey of medical practitioners was conducted from November 2020 to January 2021. Members of the EURACAN G6 Group (European Reference Network for rare adult solid cancers with focus on RAI-refractory metastatic DTC, MTC and ATC), the European Organisation for Research and Treatment of Cancer (EORTC) Endocrine Task Force and the European Thyroid Association (ETA) Cancer Group were approached to provide information relating to molecular genotyping capacities, reimbursement/fundings and treatment access for aggressive TC [6]. Here, we present published results of that survey, in addition to unpublished data. A total of 86 practitioners from 18 European Union countries (*n* = 83), Switzerland (*n* = 2) and Turkey (*n* = 1) responded (response rate ranged from 68.6% to 100%, depending on the question asked). Most of the surveyed practitioners were endocrinologists (48%) and worked in academic institutions (55%) (Table 3). Forty-seven (55%) reported being routinely involved in managing aggressive TC, 38 (81%) of whom regularly prescribed somatic molecular genotyping.

**Table 3:** Respondent characteristics in a European survey regarding molecular genotyping practices in aggressive TC [6]

Characteristics	*n* (%) among respondents (*N* = 86)
Medical specialty	
Endocrinology	41 (48%)
Oncology	24 (28%)
Pathology	4 (5%)
Nuclear Medicine	3 (3%)
Other	14 (16%)
Institution of practice	
Academic centre	47 (55%)
Cancer centre	22 (26%)
General hospital	11 (13%)
Private centre	4 (5%)
Other	2 (2%)
Routinely involved in managing aggressive TC	47 (55%)

*TC* thyroid cancer

**Fig. 1:**
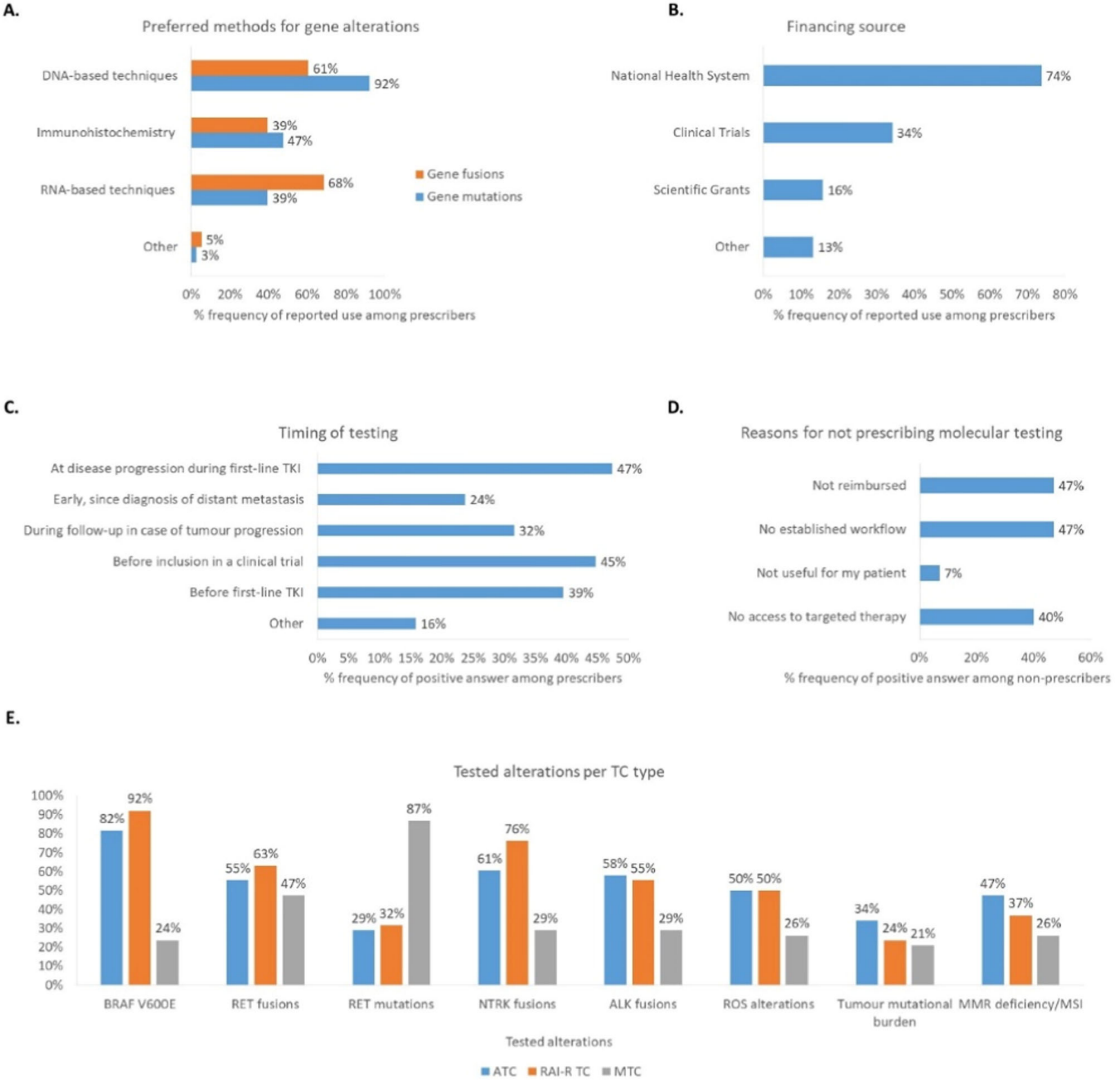
Results from a collaborative survey on molecular genotyping practices in Europe. **A** Preferred methods for the identification of gene mutations and gene fusions among practitioners who reported being routinely involved in managing aggressive TC and regularly prescribed somatic molecular genotyping (*n* = 38). **B** Source of financing for testing, as reported by prescribers (*n* = 38). **C** Timing of testing during the disease course among prescribers (*n* = 38). **D** Main reasons for not prescribing molecular testing among practitioners who were routinely involved in the management of aggressive disease but were not prescribing molecular testing (*n* = 9). **E** The most frequently investigated molecular alterations by disease type among prescribers (*n* = 38). ATC anaplastic thyroid cancer, MSI microsatellite instability, MTC medullary thyroid cancer, RAI-R radioiodine-refractory, TC thyroid cancer, TKI tyrosine kinase inhibitor

Among those regularly prescribing molecular genotyping, the preferred genotyping methods were tumour DNA-based techniques for gene mutations (92%) and RNA-based techniques for gene fusions (68%), and testing was mainly reimbursed by national healthcare systems (74%) (Fig. 1A, B). The timing of testing during the disease course varied among respondents who regularly prescribed molecular genotyping (Fig. 1C). Among the nine practitioners who were routinely involved in managing aggressive TC but did not prescribe molecular analyses, the main reasons were lack of reimbursement (47%), lack of established workflow (i.e. access to a laboratory facility performing these tests) (47%) and lack of access to targeted therapies (40%) (Fig. 1D). Of note, the latter nine respondents clustered within certain countries: Bulgaria, Greece, Lithuania, Poland and the Republic of North Macedonia. A molecular tumour board was in place for only 63% of practitioners who were routinely involved in aggressive TC management.

The most frequently investigated somatic molecular alterations were: (i) *BRAF V600E* mutation, *NTRK* fusions and *RET* fusions in RAI-R DTC; (ii) *BRAF V600E* mutation, *NTRK, ALK* and *RET* fusions in ATC; and (iii) *RET* mutations in MTC (Fig. 1E). Particularly for MTC, 87% of the practitioners who regularly prescribed molecular genotyping tested *RET* mutations, with 47% testing RET fusion alterations as well, even though it is not expected that any would be found. This suggests that there may be confusion about the alterations that can be found in MTC vs non-MTC. Access to targeted therapies was mainly in the context of clinical trials, with routine access being available in only 40% of the institutions. Overall, results of this survey confirm that even specialists treating aggressive TC across Europe may not have equal access to molecular somatic screening and/or targeted therapies. Furthermore, considering that most respondents in this survey worked in academic institutions, where access to genetic testing is expected to be easier than in the public sector, the actual uptake of molecular genotyping in TC may be lower in everyday practice.

## Discussion

Altogether, it becomes apparent that there is a large variation in Europe regarding genetic testing in TC. This variation probably reflects lack of clear testing guidelines, which in several cases is related to lack of evidence, but also practical barriers to clinical management and molecular testing implementation. The results of the abovementioned survey are particularly revealing given that the survey investigated the practices of physicians who routinely manage aggressive TC, usually in academic settings [6].

Even in such a selected group of practitioners, nearly one out of five was not prescribing molecular testing at any time during the disease course, mainly due to lack of reimbursement but also due to lack of access to a laboratory facility performing these tests. Importantly, some practitioners would not prescribe molecular testing because their patients had no access to targeted treatment, thus highlighting not only inequities in drug availability across European countries, but also a current lack of formal EMA approval in the case of dabrafenib/trametinib combination and RET inhibitors for ATC [12]. The situation is different in the USA where, for example, the dabrafenib/trametinib combination was approved in 2018 by the FDA based on the results of a basket trial including 16 patients with ATC [31]. Pralsetinib, a selective RET inhibitor, was approved by the FDA in 2020 for RET-altered TCs, but the pharmaceutical company withdrew the application to the EMA in November 2022, thus reducing the opportunities for treatment of European patients. Among prescribers, practices were variable regarding both the techniques used and the timing of the testing, which probably reflects the different amounts of available tissue, the distinct availability of referral facilities, the turnaround times and the cost/reimbursement issues. In other words, practitioners’ choice of what, how and when to test is inevitably influenced by the expertise of the available laboratory, the financing source and the existence of potential facilitators, such as access to a clinical trial. Of note, lack of awareness of biomarker alteration and testing methods could represent a real-world issue, especially for patients with non-MTC, who are treated in peripheral hospitals and/or by multiple physician specialties (e.g. endocrinologists and medical oncologists). That said, even within the survey, testing RET fusions in MTC has been reported by practitioners regularly prescribing molecular genotyping; this might reflect lack of awareness considering that RET fusions in MTC exist in literature only as case reports [32]. In support, a recent international study among pathologists from Germany, the UK, Japan and the USA reported considerable knowledge and skill gaps in *RET* testing for lung cancer and TC [33, 34]. It is also worth mentioning that half of the surveyed practitioners could not consult an institutional tumour board.

A roundtable discussion organised by the European Alliance for Personalised Medicine (EAMP) in 2022 assessed challenges in TC management related to both practitioners and the institution [35]. Indeed, on the ‘demand’ side, the forum underlined the lack of governance in the sense that international guidelines present discordances, and clinical practice is not standardised. Low awareness about TC management, both public and among clinicians, was also highlighted, as was the lack of a supportive clinical trial framework. On the ‘supply’ side, the panel raised issues of unequal access to infrastructure (particularly NGS), therapies and expertise across Europe, together with the insufficient provision of multidisciplinary forums that would facilitate collaboration among the wide range of specialists involved in TC management. Inequities in access to biomarker molecular testing across European countries were also highlighted in a recent survey on behalf of the International Quality Network for Pathology, the European Cancer Patient Coalition and the European Federation of Pharmaceutical Industries and Associations, which reported a highly variable uptake of NGS (range 0% to >50%) [36]. Similarly, a recent ESMO survey on the availability of biomolecular technologies in oncology in Europe showed that comprehensive NGS panels were mostly accessible within a clinical/translational research context, due to barriers relating to financial reimbursement of genomic tests or approved medicines and ability to prescribe a targeted agent [13].

That notwithstanding, evidence is urgently needed to address open questions. An example would be the appropriate timing of testing. Some experts have put forward a rational suggestion, which is to test as soon as the tumour is classified as aggressive (PDTC and ATC at diagnosis and DTC at time of RAI-refractoriness or progression); however, relevant data are largely missing [37]. Also, clonal dynamics during the disease course represent an uncharted area. The possibility that new molecular alterations are acquired during disease progression cannot be excluded; thus, it remains unclear whether paraffin blocks from the initial operation (diagnostic) are still informative in the case of disease progression or if a new biopsy should be promoted. Liquid biopsy is a promising alternative approach, but data regarding its technical performance in TC are scarce [30]. Regarding the testing methodology, it seems that a comprehensive NGS panel is preferred and should become available and reimbursed. In addition, the impact of NGS on the budget seems to be mitigated when the number of patients and the number of molecular alterations tested are increased [26]. Central referral laboratories could represent a working solution for ensuring access to infrastructure; however, turnaround time needs to be carefully weighed, especially for highly aggressive TC.

## Future directions

Genomic technologies require financing, laboratory infrastructure and highly trained personnel, a lack of which hamper the routine use of these technologies in clinical practice even in high-income countries, yet their value for advancing public health is increasingly being appreciated. The WHO Science Council, set up in 2021, has launched a report calling for equitable expansion of genomics to boost the health and wealth of the population worldwide [38]. At the same time, multiple stakeholders in Europe are working on a common agenda to provide broader access to high-quality oncology biomarker testing. An opportunity presents where TC scientific societies could collaborate with initiatives such as the Beating Cancer Plan and the Cancer Mission of the European Union (EU), the European Cancer Patient Coalition (ECPC) and the European Federation of Pharmaceutical Industries and Associations (EFPIA) to formulate a common EU policy on TC molecular diagnostics. It is important that national guidelines are regularly updated to incorporate new knowledge, align with the European/international recommendations and facilitate the availability of essential diagnostics and novel therapies. Oncology molecular diagnostics is a rapidly evolving field; therefore, low awareness among clinicians should be counteracted by the existence of institutional or even centralised molecular tumour boards. Finally, prospective clinical trials should be supported by both national and pharmaceutical stakeholders in European countries, especially in institutions providing care for patients with refractory TC, to produce high-quality, convincing evidence that can help drive TC management and bridge the Atlantic gap in drug access.
